# Supplementary figures and images for: The combined tumour-based Fascin/Snail and stromal periostin reveals the effective prognosis prediction in colorectal cancer patients
Source: PLoS One. 2024 Jun 27;19(6):e0304666. doi: 10.1371/journal.pone.0304666 (PMC11210851; doi:10.1371/journal.pone.0304666)

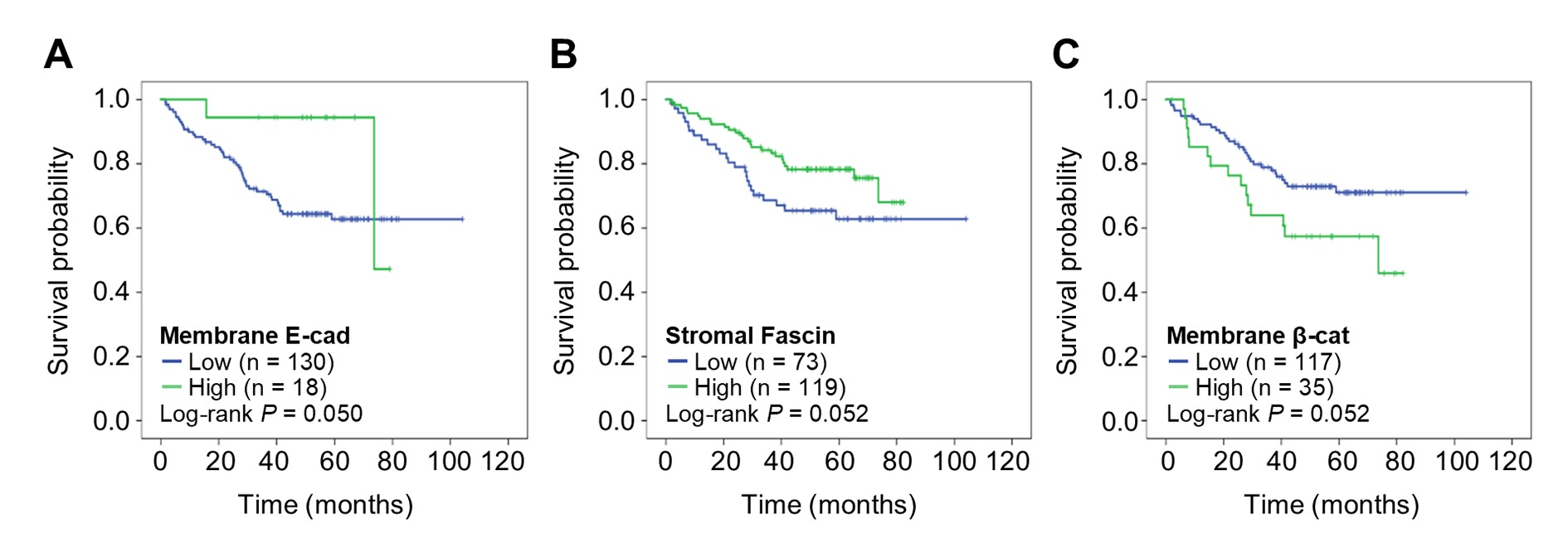

Supplement: S1 Fig — (TIF) [file pone.0304666.s001.tif]

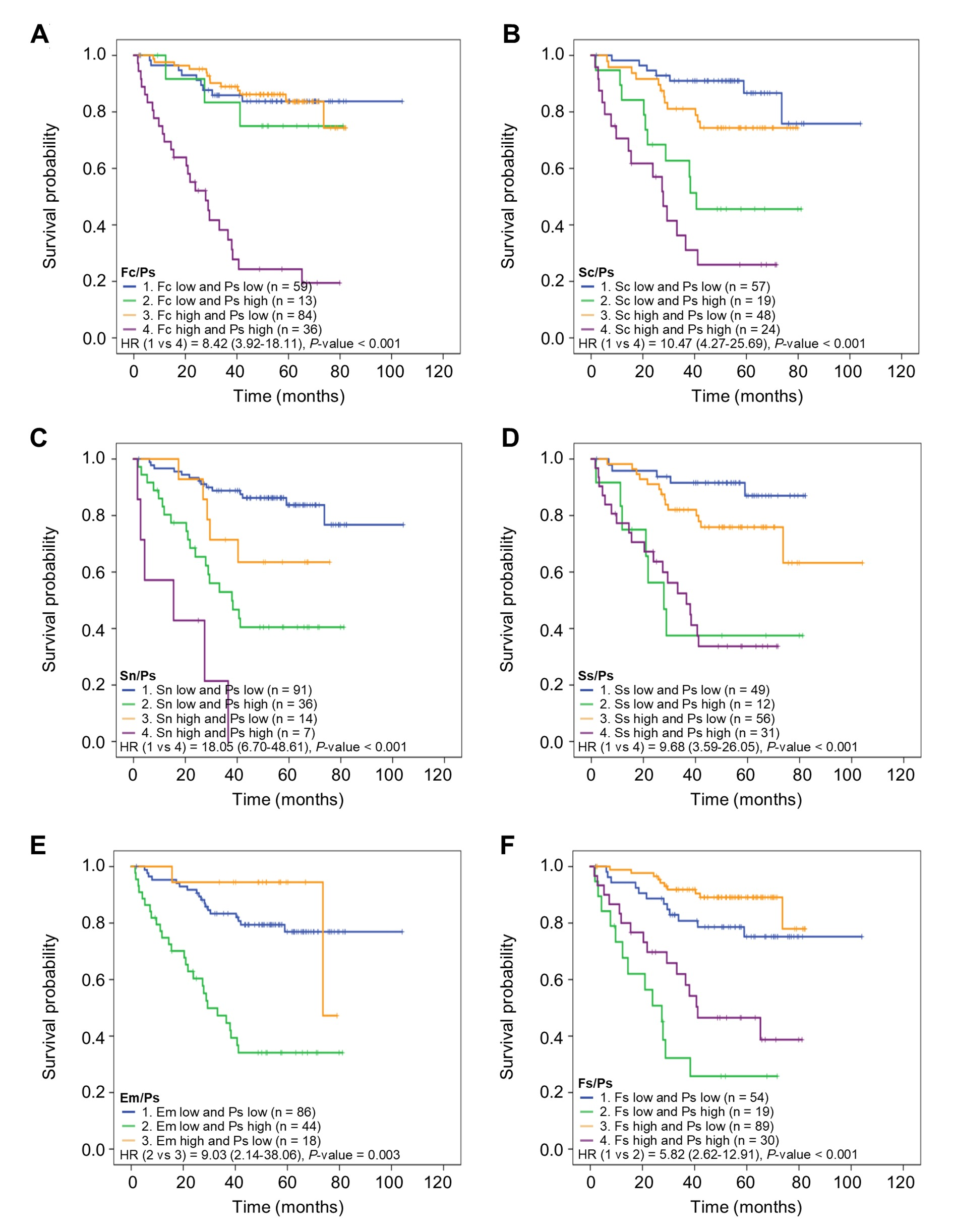

Supplement: S2 Fig — (TIF) [file pone.0304666.s002.tif]
